# Beneficial effects of curcumin and capsaicin on cyclophosphamide-induced premature ovarian failure in a rat model

**DOI:** 10.1186/s13048-018-0409-9

**Published:** 2018-04-26

**Authors:** Rauf Melekoglu, Osman Ciftci, Sevil Eraslan, Asli Cetin, Nese Basak

**Affiliations:** 1 Department of Obstetrics and Gynecology, University of Inonu, Faculty of Medicine, 44280 Malatya, Turkey; 2 Department of Medical Pharmacology, University of Inonu, Faculty of Medicine, 44280 Malatya, Turkey; 3 Elbistan State Hospital, Department of Obstetrics and Gynecology, 46300 Kahramanmaras, Turkey; 4 Faculty of Medicine, Department of Histology, University of Inonu, 44280 Malatya, Turkey; 5 Faculty of Pharmacy, Department of Pharmaceutical Toxicology, University of Inonu, 44280 Malatya, Turkey

**Keywords:** Capsaicin, Curcumin, Cyclophosphamide, Premature ovarian failure

## Abstract

**Background:**

In recent years, cancer rates have been rising among reproductive-age women. Thus, chemotherapy exposure has become an important cause of premature ovarian failure (POF). There has been growing interest regarding the preservation and restoration of ovarian function before and after oncological treatment because of the reproductive risk of chemotherapeutics and improved long-term survival of cancer patients. In this study, we sought to analyze the effects of curcumin (CRC) and capsaicin (CPS) on cyclophosphamide-induced POF in a rat model.

**Methods:**

POF in rats was induced by intraperitoneal injection of 200 mg/kg cyclophosphamide on day 1 and then 8 mg/kg/day for the following 14 days. After 14 days of cyclophosphamide administration, rats were randomly divided into three groups as follows (*n* = 10/group): POF, POF + CRC (100 mg/kg/day), and POF + CPS (0.5 mg/kg/day) to determine the effects of CRC and CPS on the cyclophosphamide-induced POF rat model. Biochemical, hormonal, and histopathological evaluations were performed on blood and tissue samples 14 days after the CRC and CPS treatments.

**Results:**

Malonaldehyde levels were significantly reduced, and glutathione levels and superoxide dismutase activity were significantly increased, in ovarian tissues in the POF + CRC and POF + CPS groups compared with the POF group. In the POF group, we observed hemorrhage and prominent mononuclear cell infiltration beneath the germinative epithelium, vascular congestion in ovarian stroma, hemorrhage around the corpus luteum, and atresia in ovarian follicles. This histopathological damage was significantly improved by treatment with CRC and CPS. There was a significant reduction in serum follicle-stimulating hormone and luteinizing hormone levels in rats treated with CRC and CPS compared with the POF group. Moreover, the levels of estradiol and anti-mullerian hormone in rats treated with CRC and CPS were significantly increased compared with the control group.

**Conclusions:**

In conclusion, CRC and CPS treatment of rats with cyclophosphamide-induced POF had a beneficial effect on reducing ovarian damage by improving tissue oxidative stress marker levels, ovarian reserve marker levels, and histopathological parameters. The significant improvements in ovarian tissue histopathological damage and hormonal levels detected in this study indicate that treatment with CRC or CPS might be a conservative treatment approach for cyclophosphamide-induced POF.

## Background

The cessation of ovarian function accompanied by increased follicle-stimulating hormone (FSH) and decreased estradiol (E2) levels in women under 40 years of age is defined as premature ovarian failure (POF) [1]. Ovarian atrophy leads to a reduced follicle reserve, which leads to menstrual irregularities, ovarian dysfunction, and infertility. Although genetic defects, autoimmune diseases, and toxic agents have been implicated in the etiology, in most cases, the cause remains unknown [2, 3]. In recent years, cancer rates have been rising among reproductive-age women. Thus, chemotherapy exposure has become an important cause of POF [4]. Cyclophosphamide (CYC) is an alkylating chemotherapeutic agent that has a detrimental effect on female reproductive organs. The reversibility of ovarian dysfunction due to CYC exposure is dependent on several factors, such as patient age, ovarian reserve, and the degree and duration of therapy [5]. There has been growing interest regarding the preservation and restoration of fertility before and after oncological treatment considering the reproductive risk of chemotherapeutics and improved long-term survival of cancer patients [6]. There are several clinical tests involving biochemical measurements and ovarian imaging that are used as ovarian reserve tests to predict reproductive potential. These biochemical tests include basal measurements of follicle-stimulating hormone (FSH), E2, inhibin B, and anti-mullerian hormone (AMH) as direct measures of the follicular pool [7].

Curcumin (CRC) and capsaicin (CPS) are naturally occurring phytochemicals that are present in two widely used food additives in Asia, turmeric and hot peppers, respectively [8]. Both of these dietary compounds have been found to possess significant health benefits as analgesics, anti-cancer agents, and anti-inflammatory agents [9, 10]. The mechanisms underlying these health effects have been attributed especially to their anti-inflammatory effects, which involve modification of macrophage function by decreasing production of proinflammatory mediators, reactive oxygen species, the metabolites of arachidonic acid, proteases, and lysosomal enzymes [11].

In this study, we sought to investigate the potential therapeutic effects of CRC and CPS on CYC-induced POF in a rat model.

## Methods

### Study protocol

The experiments were performed according to the animal ethics guidelines of the Inonu University Institutional Animals Ethics Committee (2016/A-87). In total, 40 healthy adult female Wistar albino rats (aged 3–4 months) obtained from The Experimental Animal Institute (Malatya, Turkey) were used in the experiment. Animals were housed in sterilized polypropylene rat cages under a 12/12-h light/dark cycle at an ambient temperature of 21 °C. Food and water were provided ad libitum. Rats were randomly divided into two groups to establish the chemotherapy-induced POF rat model: the control (*n* = 10) and POF groups (*n* = 30). POF was induced in the rats by intraperitoneal injection of 200 mg/kg CYC (Endoxan, EIP ECZACIBASI, Istanbul, Turkey) on day 1 and then 8 mg/kg/day for the following 14 days. After 14 days, to determine the effects of CRC and CPS on CYC-induced POF, the POF group was randomly divided into three subgroups (*n* = 10 per subgroup): POF, POF + CRC (100 mg/kg), and POF + CPS (0.5 mg/kg). The doses of CRC and CPS were chosen based on previous studies [12, 13]. The control group was given no treatment. The animals were euthanized without pain or distress using increasing anesthesia doses (intraperitoneal lethal doses of pentobarbital; Bioveta Inc., Ankara, Turkey) after the treatment course, and the ovaries were removed for histopathological analyses. Blood samples were collected under anesthesia from the left ventricle using an injector. Tissues samples were stored at − 45 °C until analyzed.

### Biochemical assays

Tissues were homogenized using a Teflon glass homogenizer in 150 mM KCl (pH 7.4) at a 1:10 (*w*/*v*) dilution of the whole homogenate. The homogenates were centrifuged at 18,000×*g* and 4 °C for 30 min to determine the malonaldehyde (MDA) and reduced glutathione (GSH) concentrations and the superoxide dismutase (SOD) and catalase (CAT) activities or at 25,000×*g* for 50 min to determine the glutathione peroxidase (GPx) activity.

### Hormonal assays

E2, FSH, luteinizing hormone (LH), and AMH were quantitatively estimated in rat serum samples using enzyme-linked immunosorbent assay (ELISA) kits (catalog numbers: SL0268Ra, SL0297Ra, SL1093Ra, and SL0504Ra, respectively; Sunlong Biotech Co., Ltd., Zhejiang, China).

### Histopathological examinations

For light microscopic evaluation, ovarian tissues were fixed in 10% formalin and embedded in paraffin wax. Paraffin wax-embedded specimens were cut into 5 μm thick sections, mounted on slides, and stained with hematoxylin and eosin (H-E). The tissue samples were examined using a Leica DFC280 light microscope and a Leica Q-Win Image Analysis system (Leica Micros Imaging Solutions Ltd., Cambridge, UK). Histopathological examination of the tissue damage was performed regarding each parameter, such as hemorrhage around the corpus luteum, vascular congestion in the ovarian stroma, hemorrhage, prominent mononuclear cell infiltration beneath the germinative epithelium and follicular atresia. At least five microscopic regions were examined to score the specimens semiquantitatively. Each sample was scored for each criterion using a scale ranging from 0 to 3 (0, none; 1, mild; 2, moderate; 3, severe). Total scores were calculated according to these parameters.

### Statistical analysis

All values are presented as means ± standard deviation. Differences were considered significant at *p* < 0.01. The SPSS software (ver. 18.0; SPSS Inc., Chicago, IL, USA) was used for the statistical analyses. The biochemical values were analyzed using one-way ANOVA and post hoc Tukey’s honestly significant difference test. Histological results were compared using Kruskal-Wallis variance analysis. When differences among the groups were detected, group means were compared using the Mann-Whitney U-test.

## Results

### Biochemical results

Antioxidant (SOD, CAT, GPx, and GSH) and oxidant parameters (MDA) in rat ovaries are presented in Table 1. MDA levels were significantly decreased, whereas GSH levels and SOD activity were significantly increased, in ovarian tissues in the POF + CRC and POF + CPS groups compared with the POF group. GPx levels and CAT activities were similar among the groups.

**Table 1:** Levels of MDA, GSH, SOD, GPx, and CAT in rat ovarian tissue

Groups	MDA (nmol/g tissue)	GSH (nmol/ mL)	SOD (U/mg protein)	GPx (U/mg protein)	CAT (kU/ mg protein)
Control	5.15 ± 1.29^a^	47.4 ± 2.77^a^	97.5 ± 15.1^a^	788.1 ± 103.2^a^	0.009 ± 0.003^a^
POF	8.66 ± 1.30^b^	39.4 ± 3.11^b^	79.3 ± 11.2^b^	739.7 ± 118.5^a^	0.006 ± 0.002^a^
POF + CRC	3.07 ± 1.35^c^	49.0 ± 5.24^a^	105.5 ± 16.1^a^	692.8 ± 114.2^a^	0.012 ± 0.007^a^
POF + CPS	3.24 ± 0.51^c^	48.8 ± 3.22^a^	109.9 ± 12.7^a^	719.3 ± 103.8^a^	0.011 ± 0.005^a^

*MDA: malonaldehyde; *GSH*: glutathione; *SOD*: superoxide dismutase; *GPx:* glutathione peroxidase; *CAT*: catalase; *POF*: premature ovarian failure; *CRC*: curcumin; *CPS:* capsaicin

*Mean values bearing different superscript letters within the same column are significantly different (p < 0.01)

### Histological results

**Fig. 1:**
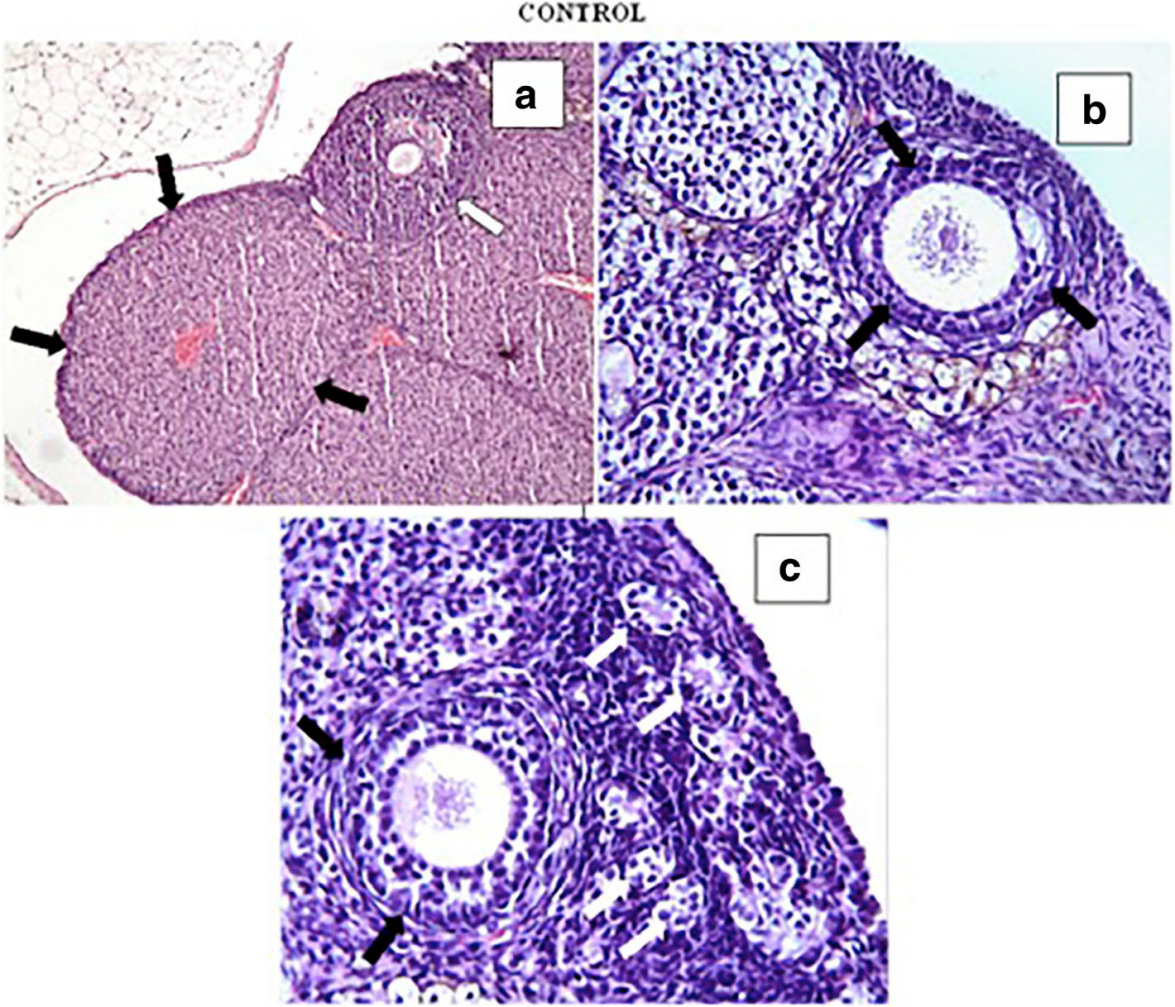
A normal histological appearance was observed in the control group. Corpus luteum (black arrows) (**a**), secondary follicles (white arrow) (**a**), multilaminary primary follicles (black arrows) (**b**, **c**), primordial follicles (white arrows) (**c**) were observed in the control group (**a**: H-E, × 10; **b** and **c**: H-E, × 40)

**Fig. 2:**
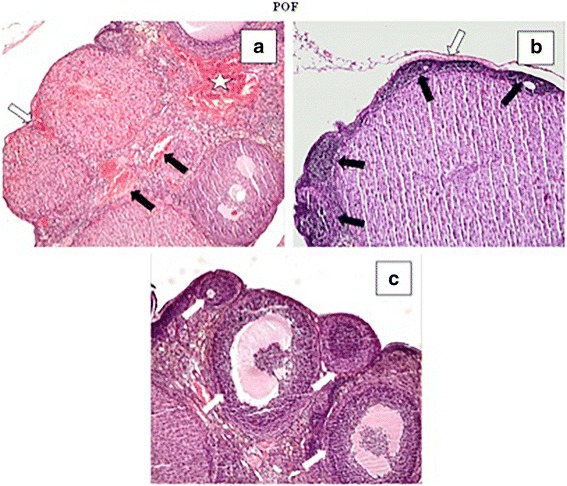
In the POF group, we detected vascular congestion (black arrows) (**a**), hemorrhage around the corpus luteum (white arrow) and ovarian stroma (white star), follicular atresia (**c**) (white arrows), hemorrhage and mononuclear cell infiltration beneath the germinative epithelium (**b**) and ovarian stroma (**c**). A: H-E, × 4; B and C: H-E, × 10)

**Fig. 3:**
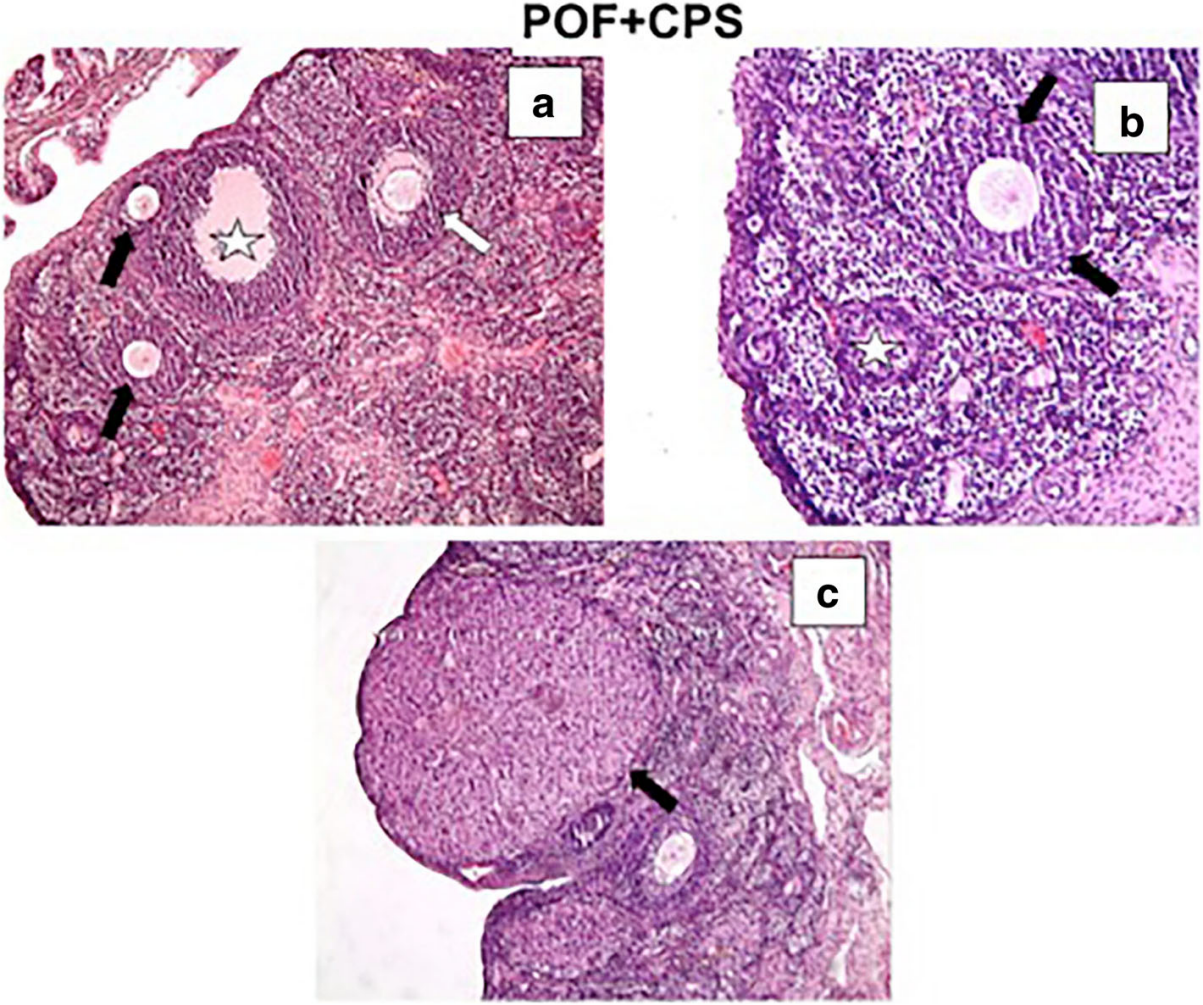
In the POF + CPS group, we detected decreased vascular congestion, hemorrhage, and mononuclear cell infiltration (**a**–**c**). The corpus luteum structure was normal (**c**). Atretic follicles (black stars) (**a**, **b**), multilaminary primary follicles (black arrows), and secondary follicles (white arrow) were observed in **a** and **b**. A and C: H-E, × 10; B: H-E, × 20

**Fig. 4:**
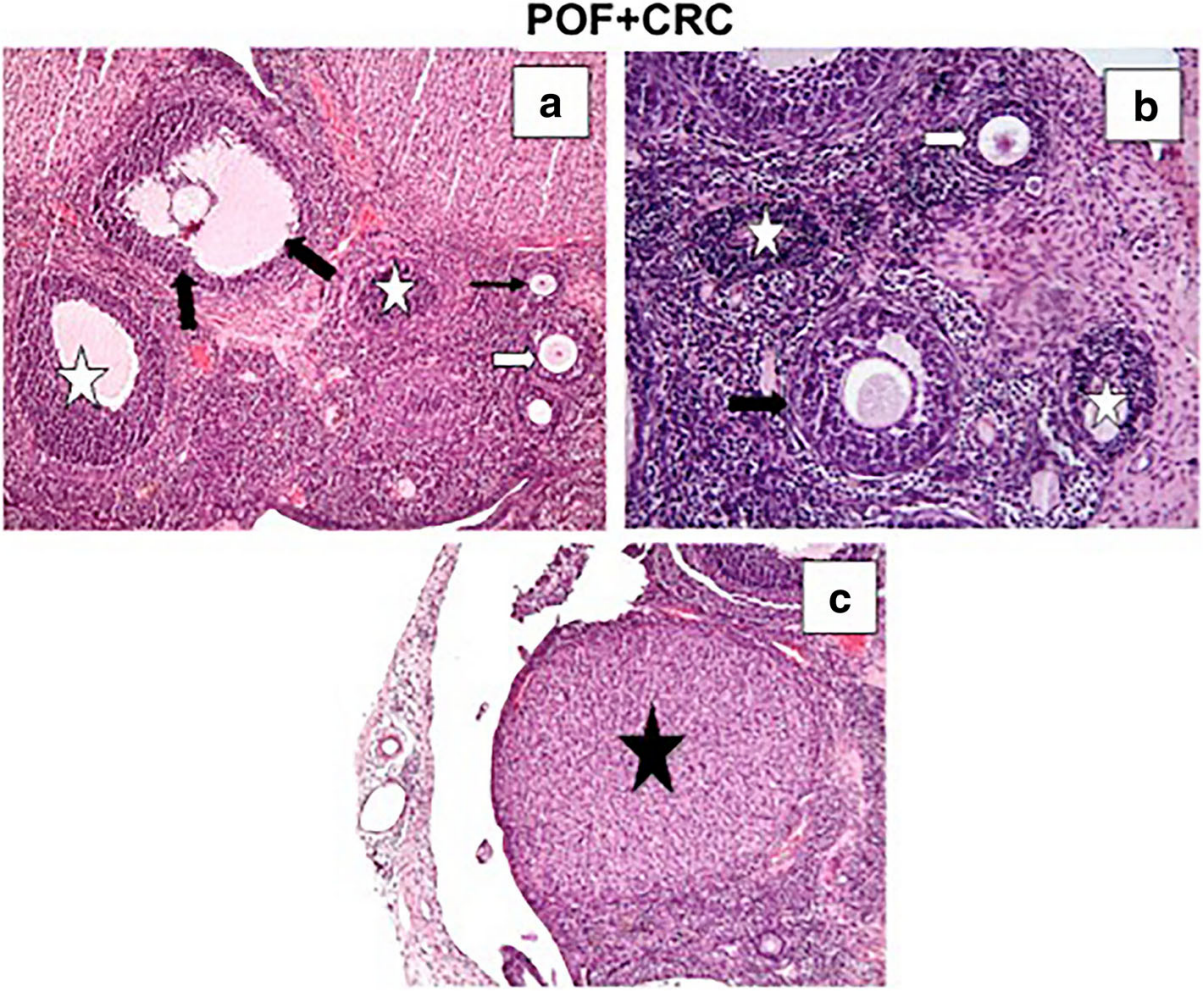
An improvement in histological appearance was apparent in the POF + CRC group. Decreased vascular congestion (**a**) and atretic follicles (white stars) (**a**, **b**) were observed. A Graffian follicle (**a**), unilaminary primary follicle (thin black arrow) (**a**), multilaminary primary follicle (white arrow) (**a**, **b**), secondary follicle (black arrow), and corpus luteum (black star) (**c**) were seen in the POF + CRC group. A and C: H-E, × 10; B: H-E, × 20

In the control group, ovarian tissues showed a normal histological appearance. The secondary follicles (Fig. 1a), multilaminary primary follicles (Fig. 1b and c), primordial follicle (Fig. 1c), and corpus luteum exhibited normal appearances. In the POF group, we observed hemorrhage around the corpus luteum (Fig. 2a), vascular congestion in the ovarian stroma (Fig. 2a), and hemorrhage and prominent mononuclear cell infiltration beneath the germinative epithelium (Fig. 2b). In addition, some atretic ovarian follicles (Fig. 2c) were found in the POF group. We showed that all histological parameters were significantly improved after administration of CPS and CRC. We observed normal multilaminar primary follicles, secondary follicles (white arrow) (Fig. 3a and b), and corpus luteum structures (Fig. 3c) in the POF + CPS group. Decreased vascular congestion (Fig. 4a), fewer atretic follicle (Fig. 4a and b), and normal Graffian follicles, unilaminary primary follicles, multilaminary primary follicles (Fig. 4a and b), secondary follicles, and corpus luteum (Fig. 4c) were detected in the POF + CRC group compared with the POF group. Histopathologic scores for all four groups are demonstrated in Table 2. A significant increment in vascular congestion, hemorrhage, mononuclear cell infiltration and follicular atresia was observed in the POF group compared with the other groups. All histological parameters were significantly improved with the administration of CRC and CPS. However, there were no significant differences between POF + CRC and POF + CPS groups regarding the histopathological scores.

**Table 2:** Histopathological score of groups

Groups	Histopathologic Damage (Mean ± SD)
Control	0.43 ± 0.09^a^
POF	1.98 ± 0.10^b^
POF + CRC	1.25 ± 0.15^c^
POF + CPS	1.38 ± 0.11^c^

*POF: premature ovarian failure; *CRC*: curcumin; *CPS*: capsaicin

*Mean values bearing different superscript letters within the same column are significantly different (*p* < 0.01)

### Hormonal assessment

Serum FSH and LH levels were significantly reduced in rats treated with CRC and CPS compared with the POF group. However, E2 and AMH levels were significantly increased in rats treated with CRC and CPS compared with the POF group. However, there was no significant difference in serum hormone levels between the POF + CRC and POF + CPS groups. The effects of CRC and CPS on the ovarian reserve markers are shown in Table 3.

**Table 3:** Hormone levels in each group

Groups	E2 (ng/mL)	FSH (ng/mL)	LH (ng/mL)	AMH (ng/mL)
Control	81.5 ± 4.91^a^	5.07 ± 0.27^a^	2.84 ± 0.47^a^	7.03 ± 0.82^a^
POF	41.5 ± 2.86^b^	8.87 ± 0.14^b^	4.39 ± 0.55^b^	3.29 ± 0.30^b^
POF + CRC	62.2 ± 6.20^c^	6.64 ± 0.46^c^	3.14 ± 0.51^a^	5.91 ± 0.45^c^
POF + CPS	67.2 ± 3.02^c^	6.43 ± 0.45^c^	3.48 ± 0.46^a^	6.56 ± 0.82^ac^

*POF: premature ovarian failure; *CRC*: curcumin; *CPS:* capsaicin, *E2*: estradiol, *FSH*: follicle-stimulating hormone; *LH:* luteinizing hormone; *AMH*: anti-mullerian hormone

*Mean values bearing different superscript letters within the same column are significantly different (*p* < 0.01)

## Discussion

In this study, we examined the ovarian protective effects of CRC and CPS in a rat model of CYC-induced ovarian insufficiency. We demonstrated that chemotherapy alone caused a marked decrease in the ovarian reserve, which was associated with increased tissue oxidative stress, impaired hormonal changes, and increased histological damage. In contrast, the tissue oxidative stress parameters, ovarian reserve markers, and histopathological changes were ameliorated significantly in rats receiving CPS and CRC concurrently with chemotherapy. Alkylating agents such as CYC, busulphan, and dacarbazine are the most ovotoxic chemotherapy drugs, creating DNA crosslinks, which in turn induce DNA breaks, ultimately triggering apoptosis [14, 15]. Roness et al. reviewed agents that prevent chemotherapy-induced ovarian damage and noted that AS-101, AMH, imatinib, sphingosine-1-phosphate, granulocyte colony-stimulating factor, bortezomib, and multidrug resistance gene-1 are effective for preventing chemotherapy-induced ovarian damage. They found different mechanisms of action associated with each protective agent, including prevention of follicle activation, anti-apoptosis effects, vascular effects, and gene upregulation [16]. To our knowledge, this is the first report to evaluate the effects of CRC and CPS on prevention of chemotherapy-induced ovarian damage, and the results suggest that both of these agents have a protective effect against CYC-induced ovarian insufficiency.

The mechanisms of the antioxidative and anti-inflammatory effects of CRC have been reported to involve reductions in inducible nitric oxide synthase and inflammatory cytokine (interleukin-1β and − 6) expression via pro-inflammatory nuclear factor kappa B (NF-κB) inhibition [17]. In addition, CRC has been demonstrated to upregulate the gene expression and enzymatic activity of detoxification enzymes such as NAD(P)H: quinone oxidoreductase 1 and glutathione S-transferase [18]. CPS has been demonstrated to exert pro-apoptotic activity by downregulating the transient receptor potential vanilloid (TRPV) receptor, as well as an inhibitory effect on NF-κB [19]. In vitro studies by Arzuman et al. reported the beneficial effect of the combination of CPS and CRC with monofunctional platinum (II) complex in platinum resistance ovarian cancer cell lines. The proposed therapeutic mechanism of CPS and CRC on cancer cells was reported to inhibit the activation of NF-κB and block the activation of signal transducer and activator of transcription 3 (induced by IL-6) [20, 21]. The results of this study also confirmed protective effects of CRC and CPS against CYC-induced ovarian insufficiency in rat ovaries by demonstrating CRC and CPS antioxidative and anti-inflammatory effects, including reduced lipid peroxidation, increased antioxidant activity, and improved histological parameters.

MDA is an end product of lipid peroxidation, and increased MDA levels reflect oxidative stress. In contrast, increased levels of GSH and activities of SOD, CAT, and GPx indicate tissue healing after oxidative damage [22]. Oxidative stress leads to ovarian failure by inhibiting nuclear and cytoplasmic maturation of oocytes and inducing apoptosis [23]. In this study, we demonstrated significant improvements in tissue oxidative stress markers, including MDA, GSH, and SOD, by CPS and CRC after chemotherapy-induced ovarian damage. Similarly, Wang et al. investigated the protective effect of CRC against oxidative stress induced by sodium arsenite. They found that CRC reduced the level of MDA and increased the activities of antioxidant enzymes, including SOD and GPx [24]. Furthermore, Qin et al. demonstrated that CRC pretreatment significantly suppressed zearalenone-induced oxidative stress by increasing the activities of SOD and CAT [25]. Consistent with this study, Park et al. demonstrated a protective effect of CPS against testicular injury induced by scrotal hyperthermia [26]. They showed that CPS pre-treatment significantly suppressed oxidative stress (levels of MDA, phospholipid hydroperoxide glutathione peroxidase, heat shock 70-kDa protein 1, and manganese superoxide dismutase) and apoptosis induced by heat stress in testes.

The results of this study also revealed that CRC and CPS improved ovarian reserve markers after CYC-induced ovarian insufficiency. Significant increases in AMH and E2 levels and significant decreases in FSH and LH levels were found in the CPS- and CRC-treated groups compared with the POF group. Few experimental studies have evaluated the effects of chemotherapy-induced ovarian damage and antioxidants on ovarian reserve markers. Özcan et al. evaluated the effects of resveratrol against cisplatin-induced oxidative damage of the ovarian reserve in rats. They found that resveratrol significantly increased the AMH level compared with the control group [27]. The improvements in ovarian reserve markers observed in this study suggest that CRC and CPS have beneficial effects on ovarian function restoration after chemotherapy exposure.

In the present study, we showed that CRC and CPS treatments improved histological parameters, such as hemorrhage, vascular congestion, and mononuclear cell infiltration, in ovarian tissue exposed to CYC treatment. Czekaj et al. investigated the effect of CRC on the protection of gastric mucosa against stress-induced gastric mucosal damage and demonstrated that the number of experimental stress-induced gastric lesions was markedly reduced by CRC pretreatment [28]. Wang et al. showed beneficial effects of CPS in a retinal ischemia–reperfusion mouse model, in that retinal ischemia–reperfusion damage (especially the numbers of astrocytes and microglia/macrophages) was significantly improved via the release of endogenous somatostatin [29]. The mechanism by which CRC and CPS protect ovarian tissues may involve reduced exposure to oxidative injury and decreased stimulation of TRPV receptors, which have been proposed to possess antioxidant and anti-inflammatory activities [30].

## Conclusions

In conclusion, the treatment of CYC-induced POF with CRC and CPS had a beneficial effect on reducing ovarian damage by improving tissue oxidative stress markers, ovarian reserve markers, and histopathological parameters. The significant improvement in tissue oxidative stress parameters, histopathological damage to ovarian tissue, and hormonal levels detected in this study indicate that treatment with CRC or CPS may be a conservative treatment approach for CYC-induced POF.

## Abbreviations

AMH: Anti-mullerian hormone
CAT: Catalase
CPS: Capsaicin
CRC: Curcumin
CYC: Cyclophosphamide
E2: Estradiol
FSH: Follicle-stimulating hormone
GPx: Glutathione peroxidase
GSH: Glutathione
LH: Luteinizing hormone
MDA: Malonaldehyde
NF-κB: Nuclear factor kappa B inhibition
POF: Premature ovarian failure
SOD: Superoxide dismutase
TRPV: Transient receptor potential vanilloid
